# Detection of Type, Blended Ratio, and Mixed Ratio of Pu’er Tea by Using Electronic Nose and Visible/Near Infrared Spectrometer

**DOI:** 10.3390/s19102359

**Published:** 2019-05-22

**Authors:** Sai Xu, Xiuxiu Sun, Huazhong Lu, Qianqian Zhang

**Affiliations:** 1 Public Monitoring Center for Agro-Product of Guangdong Academy of Agricultural Sciences, Guangzhou 510640, China; xusai@gdaas.cn; 2 Guangdong Academy of Agricultural Sciences, Guangzhou 510640, China; 3 Indian River Research and Education Center, University of Florida, Ft. Pierce, FL 34945, USA; sunxiuxiu6@gmail.com; 4 College of Engineering, South China Agricultural University, Guangzhou 510640, China; 664379505qian@stu.scau.edu.cn

**Keywords:** Pu’er tea, quality, electronic nose, visible/near infrared spectrometer, detection, convolutional neural network

## Abstract

The objective of this study was to find an intelligent and fast method to detect the type, blended ratio, and mixed ratio of ancient Pu’er tea, which is significant in maintaining order in the Pu’er tea industry. An electronic nose (E-nose) and a visible near infrared spectrometer (VIS/NIR spectrometer) were applied for tea sampling. Feature extraction was conducted using both the traditional method and a convolutional neural network (CNN) technique. Linear discriminant analysis (LDA) and partial least square regression (PLSR) were applied for pattern recognition. After sampling while using the traditional method, the analysis of variance (ANOVA) results showed that the mean differential value of each sensor should be selected as the optimal feature extraction method for E-nose data, and raw data comparison results showed that 19 peak/valley values and two slope values were extracted. While the format of E-nose data was in accord with the input format for CNN, the VIS/NIR spectrometer data required matrixing to meet the format requirements. The LDA and PLSR analysis results showed that CNN has superior detection ability, being able to acquire more local features than the traditional method, but it has the risk of mixing in redundant information, which can act to reduce the detection ability. Multi-source information fusion (E-nose and VIS/NIR spectrometer fusion) can collect more features from different angles to improve the detection ability, but it also contains the risk of adding redundant information, which reduces the detection ability. For practical detection, the type of Pu’er tea should be recognizable using a VIS/NIR spectrometer and the traditional feature extraction method. The blended ratio of Pu’er tea should also be identifiable by using a VIS/NIR spectrometer with traditional feature extraction. Multi-source information fusion with traditional feature extraction should be used if the accuracy requirement is extremely high; otherwise, a VIS/NIR spectrometer with traditional feature extraction is preferred.

## 1. Introduction

Yunnan province of China is the heartland and presumed sourceof tea in the world [1]. The famous Pu’er tea leaves are plucked from the trees, named Daye in Pu’er city of Yunnan province, and then put through a processing procedure before finishing [2]. Due to its unique soil and climate, tea plants will only develop the flavor in Pu’er city (aroma, liquor color, and taste), which is traditionally considered to be correct As a consequence, Yunnan Pu’er tea has been awarded a national Geographic Indication designation in China (National standard number: GB/T 22111-2008). Pu’er tea is loved by consumers all around the world because to the first-class flavor and special health efficacy, with vast market demand [3]. However, while Pu’er tea has a much higher market price than most other teas and different types of Pu’er tea vary considerably in price, the various types of Pu’er tea (as well as some non-Pu’er teas) are similar in appearance and hard to discriminate between by ordinary consumers. Thus, the problem of developing an accurate tea type detection technique is particularly important in order to maintain the integrity of the Pu’er tea industry. 

Ancient Pu’er tea is the most rare and expensive type due to its specific flavor development over hundred years of cultivation. Since most consumers cannot afford ancient Pu’er tea, two lower-cost Pu’er tea products were launched by the industry to satisfy consumer demand. These were: tableland Pu’er tea [4] and blended Pu’er tea [5]. Tableland Pu’er tea is produced by newly planted Pu’re tea trees. In contrast to ancient Pu’er tea, the flavor is much lighter and the price is much cheaper. The blended Pu’er tea is in the medium-price range, and it is a certain ratio of ancient and less expensive varieties of Pu’er tea. The flavor grade of blended Pu’er tea is between ancient and tableland Pu’er tea. Due to the large price differences among them, a black-market industry deliberately selling mislabeled tea has flourished by exploiting consumers’ inability to visually distinguish between the different varieties of Pu’er and between Pu’er and other less expensive types of tea [6,7]. The effect on the industry has been that consumers, having unknowingly purchased counterfeit Pu’er tea, feel that the quality of Pu’er tea has diminished, which thus reduces the marketability of legitimate Pu’er tea. Thus, finding a way to quickly determine a batch of Pu’er tea’s actual origination, specific blended ratio, and specific mixed ratio is a vitally important concern of the Pu’er tea industry.

Sensory evaluation [8] and physicochemical index detection [9] are two current important methods of tea quality detection. However, sensory evaluation mostly relies on human sensation, and it can be easily confounded by human subjectivity. Physicochemical index detection is complex in operation, and time and labor consuming. Thus, those two methods cannot adequately meet the needs of the Pu’er tea industry for rapid accurate detection. A more intelligent and faster method needs to be explored.

Electronic nose (E-nose) [10] and visible/near Infrared (VIS/NIR) spectrometer [11] have wide applications in food quality detection. Both the E-nose and VIS/NIR spectrometer can acquire a sample’s quality information quickly and then automatically evaluate the detection results in seconds. Previous research results showed that using an E-nose is a feasible means of detect a Pu’er tea sample’s grade within the same type [12], its storage time [13,14], and its place of storage [15]. The VIS/NIR spectrometer is an accurate method of detecting a Pu’er tea sample’s storage time [16], degree of fermentation [17], polyphenol content [18], and free amino acid content [19]. All previous researches proved that both an E-nose and a VIS/NIR spectrometer could efficiently detect and evaluate Pu’er tea’s quality information. However, whether an E-nose or a VIS/NIR spectrometer can detect a Pu’er tea sample’s type, its blended ratio, or its mixed ratio is still unknown. 

In this research, six ancient Pu’er tea types were collected for a type detection experiment, one tableland Pu’er tea was blended with an ancient Pu’er tea for a blended ratio detection experiment, and a similar looking green tea was mixed with ancient Pu’er tea for a mixed ratio detection experiment. The E-nose and VIS/NIR spectrograph were applied for sampling. The aim of this research is to develop a fast and accurate procedure to detect the type, blended ratio, and mixed ratio of a Pu’er tea sample in order to provide a superior technique for maintaining the integrity of the Pu’er tea industry.

## 2. Materials and Methods

### 2.1. Experimental Pu’er Tea

All of the experimental samples were raw tea. Minqiang Tea Industry Inc. provided ancient Pu’er tea in Lincang, Yunnan province; tableland Pu’er tea and non-Pu’er green tea were bought from a local market (Table 1). Six ancient Pu’er tea types, (Laobanzhang (LBZ), Laomane (LME), Hekai (HK), Pasha (PS), Boma (BM), and Naka (NK)), were used for the type detection experiment. Yiwu (YW) ancient tea and Yiwu (YW) tableland tea were used for the blended ratio detection experiment. Banpen (BP) ancient tea mixed with Qingliangshan (QLS) common green tea was used for the mixed ratio detection experiment. To cover the different ratios between pure ancient Pu’er tea (1:0) and pure non-ancient Puer tea (0:1), and to keep the homogeneity of the gradient space of ratios, the ratios of ancient Pu’er tea to other teas in the blended and mixed samples were 1:0, 3:1, 1:1, 1:3, and 0:1. A pulverizer grounded each sample under 29,000 r/min for 2 min. to ensure the homogeneity of blend and mix.

### 2.2. E-nose Sampling

A portable E-nose (PEN 3, AIRSENSE Inc., Schwerin, Germany) was used for the volatile feature sampling of tea samples. This E-nose is composed of a sensor array, a sampling and cleaning channel, and a data acquisition unit. The sensor array contains 10 metal oxide gas sensors that are sensitive to different type of volatiles. Table 2 shows the features of each sensor.

Each tea sample was 5 g of material, was put in 200 mL glass beaker, was sealed with a double layer of preservative film, and was stored at for 30 min. Before sampling, zero gas (room air that had been filtered through standard activated carbon) was pumped into the cleaning channel to reset the sensors. The operating parameters of the E-nose were set at a sampling interval of 1 s; flush time of 60 s; zero point trim time of 10 s; measurement time of 100 s; pre-sampling time of 5 s; and, injection flow of 240 mL/min. 

### 2.3. VIS/NIR Spectrograph Sampling

Aportable spectrometer(ASD Feildspec 3, Analytica Spectra Devices Inc., Boulder, CO, USA) was used to acquire a sample’s VIS/NIR spectrum information. The wavelength range of this spectrometer is 350 to 2500 nm. The sampling interval and spectral resolution of 350 to 1000 nm are 1.4 nm and 3 nm, respectively, and the sampling interval and spectral resolution of 1000 to 2500 nm are 2 nm and 10 nm, respectively. The spectrometer’s vegetation probe is composed of a light source (with a wavelength coverage from 350 to 2500 nm) and a fibre-optics probe, which was used to acquire the tea sample’s reflectance spectrum. 

The same samples of E-nose sampling were transferred to an opaque 5 mL beaker to compare the detection ability. When sampling, we kept the vegetation probe straight down and put it in contact with the surface of the tea sample. Each sampling was repeated 10 times, while using the average as the sampling result. Whiteboard correction was conducted before each sampling. 

### 2.4. Feature Extraction

#### 2.4.1. Traditional Feature Extraction

For E-nose data’s feature extraction, the maximum value (the maximum value of a complete response value) [20], mean differential value (the mean value of the differential of the electronic nose response curve for each sampling time) [21], average value (the average value of a complete response value) [22], and stable value (the response value after the sensor response curve turn stable, the 95th s was selected as stable value in this study) [23] methods were conducted for comparison. Analysis of variance (ANOVA) [24] was applied to evaluate the comparison, and Table 3 shows the results. For ANOVA, thesignificance level (*p* value) shows the degree of difference among groups. If the *p* is less than or equal to 0.05 this demonstrates that the difference among groups is significant, while if *p* is greater than 0.05 the difference is insignificant. The lower the *p* value, the more significant the difference. Consequently, mean differential value can be seen as having better feature extraction accuracy than the other three methods, as the mean differential value finds significant difference between different Pu’er tea types, blended ratios, and mixed ratios (*p* < 0.05).

**Figure 1:**
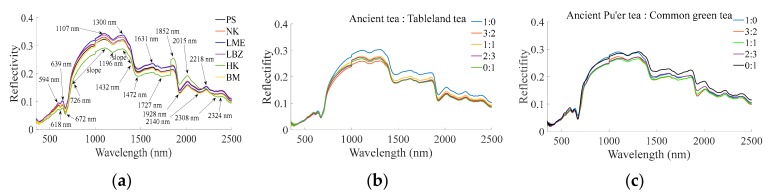
Average value comparison of tea sample in different (**a**) type; (**b**) blended ratio; (**c**) mixed ratio. The same feature extraction with (**a**) was also appliedforPu’re tea’s blended ratio and mixed ratio detection, and they were not marked in (**b**,**c**) repeatedly.

Figure 1 shows the average value ofreflectivity of each tea group. For average value ofreflectivity of different ancient Pu’er tea types (Figure 1a), the reflectivity curve shape of HK is obviously different from others throughout the entire detected wavelength, particularly at 1852 nm and from 2140 to 2308 nm. The curves of PS, NK, LME, LBZ, and BM are generally similar in shape, but differ in peak and valley levels at 594, 618, 639, 672, 726, 1107, 1196, 1300, 1432, 1472, 1631, 1727, 1852, 1928, 2015, 2140, 2218, 2308, and 2324 nm. Additionally, the increasing and decreasing rates were different from 726 to 1107 nm and from 1300 to 1432 nm, respectively. Thus, 594, 618, 639, 672, 726, 1107, 1196, 1300, 1432, 1472, 1631, 1727, 1852, 1928, 2015, 2140, 2218, 2308, and 2324 nm values and two slope values from 726 to 1107 nm and from 1300 to 1432 nm were selected as the feature values for the next Pu’er tea’s type detection. For the average value ofreflectivity for different blended (Figure 1b) and mixed ratios (Figure 1c), the overall curve shapes are similar to pure Pu’er tea (Figure 1a), (except for HK around 1852 nm and from 2140 to 2308 nm), while the actual peak/valley values are dissimilar enough to pure Pu’er tea to allow for a determination of type. Thus, the same feature extraction with Figure 1a was also appliedforPu’re tea’s blended ratio and mixed ratio detection, and they were not marked in Figure 1b,c repeatedly.

#### 2.4.2. Convolutional Neural Network Feature Extraction

**Figure 2:**
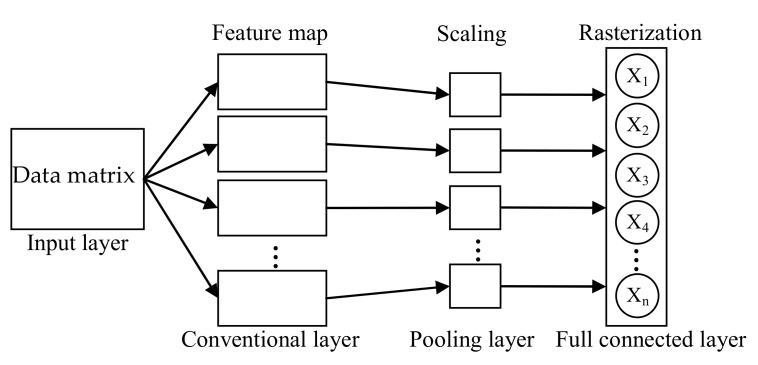
Infrastructure of a typical convolutional neural network (CNN).

The convolutional neural network (CNN) [25] has a strong ability for feature mining via convolution and pooling operations. The sparse connectivity and weight sharing of CNN allow for a better extraction of the abstract features and a certain degree of training parameter number simplifications. The infrastructure of a typical CNN contains an input layer, a convolution layer, a pooling layer, and a full connected layer, as shown in Figure 2. The input layer must be a matrix to satisfy the requirements of the convolution and pooling operations. The convolutional layers apply a convolution operation to the input while using feature maps, and then passing the result to the next layer. Each feature connects with the input layer by weight/filter. The pooling layers reduce the size of the data by combining the outputs of neuron clusters at one layer into a single neuron in the next layer. The fully connected layers connect every neuron in one layer to every neuron in another layer, and they are used for the rasterization of data after pooling.

Even though CNN would sacrifice the simplification of detection model, it is still meaningful if CNN can largely improve the detection accuracy when the detection accuracy with traditional feature extraction is unsatisfied. The strong feature extraction and expression ability of CNN was proven for image and speech signal feature extraction [26,27]; however, it has not been extensively applied for E-nose and VIS/NIR spectrum information feature extraction, as was done in this research. The shape of E-nose data for one sample in this research is a 100×10 matrix, which is suitable for the requirements of the CNN input format. Conversely, the VIS/NIR Spectrum data of one sample in this research is a vector with 2150 elements. A two-dimensional matrixing operation is needed for each sample’s VIS/NIR spectrum data in order for the VIS/NIR Spectrum data to meet the requirements of the CNN input format.

S = XX^T^ (1)

where S is the two-dimensional matrix form of one sample’s VIS/NIR Spectrum data, X is the vector form of one sample’s VIS/NIR spectrum data, and X^T^ is the transposed vector of X. However, a 2150 × 2150 matrix is excessively large and it greatly reduces the CNN operation efficiency. Therefore, dimension reduction algorithms are necessary before the two-dimensional matrixing operation. In this study, principal component analysis (PCA) [28] was applied for the dimension reduction of VIS/NIR Spectrum data. After repeated testing, it was determined that too many or too few principal components would decrease the detection accuracy, and that the first 30 principal components was the appropriate number to use for further analysis (the total contribution rate of the 30 principal component factors is close to 100%). 

After repeated training, the optimal parameters of CNN feature extraction network parameters for type, blended ratio, and mixed ratio detection were found, and they are shown in Table 4.

### 2.5. DataAnalysis Methods

Linear discriminant analysis (LDA) [29] and partial least square regression (PLSR) [30] were applied to compare the effect of different feature extraction methods and build up the Pu’er tea quality detection model. As a linear classifier, the LDA can map sample data to a two-dimensional space by dimensionality reduction and classify different sample groups. PLSR has been widely used for quality prediction due to its stable performance and fast computing speed, and was used for Pu’er tea quality prediction modeling in this research. 

## 3. Results

### 3.1. Type Detection of Ancient Pu’er Tea

#### 3.1.1. LDA Classification of Ancient Pu’erTea’s Type

**Figure 3:**
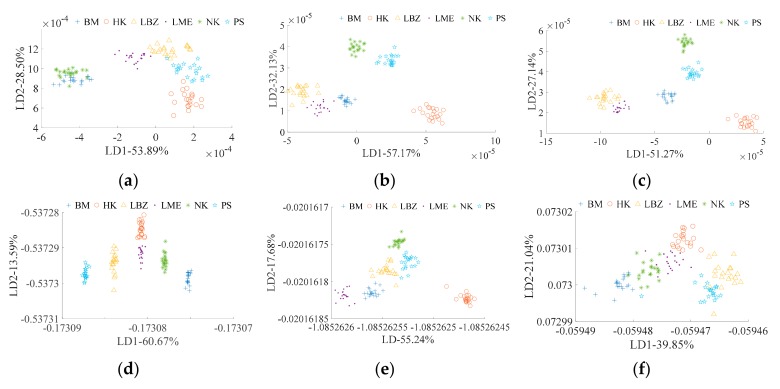
Linear discriminant analysis (LDA) for ancient Pu’er tea discrimination based on E-nose. (**a**–**c**) are traditional feature extraction; (**d**–**f**) are CNN feature extraction; (**a**,**d**) are E-nose detection; (**b**,**e**) are visible/near Infrared (VIS/NIR) spectrometer detection; and, (**c**,**f**) are multi-information fusion detection.

The LDA classification results of ancient Pu’er tea type based on E-nose and traditional feature extraction is shown in Figure 3a. All types overlap with each other, and only HK and LME can be distinguished from one another. Figure 3d shows the LDA classification results of ancient Pu’er tea type based on E-nose and CNN feature extraction. This method has better classification ability than traditional feature extraction (Figure 3a), as all types can be distinguished from one another. Figure 3b shows the LDA classification results of ancient Pu’er tea type that is based on VIS/NIR spectrometer and traditional feature extraction, and all types can be classified. The LDA classification result of ancient Pu’er tea’s type based on VIS/NIR spectrometer and CNN feature extraction is shown in Figure 3e. All types can be classified; however, the distances between the type groups using this method (shown in Figure 3e) are smaller than when using the traditional feature extraction method (shown in Figure 3b. The LDA classification results of ancient Pu’er tea type based on multi-source information fusion (E-nose and VIS/NIR spectrometer data fusion) and traditional feature extraction is shown in Figure 3c; all types can be classified. Figure 3f ahows the LDA classification results of ancient Pu’er tea type that is based on fusion information and CNN feature extraction; all types cannot be classified. For traditional feature extraction, both the VIS/NIR spectrometer and multi-information fusion can efficiently classify Pu’er tea types, but multi-information fusion did not significantly alter the classification ability of the VIS/NIR spectrometer. For CNN feature extraction, multi-information fusion reduced the classification ability of single classification methods. Therefore, VIS/NIR spectrometer with traditional feature extraction and E-nose with CNN feature extraction are the optimal ways of detecting the Pu’er tea type.

#### 3.1.2. PLSR Prediction of Ancient Pu’erTea’s Type

**Figure 4:**
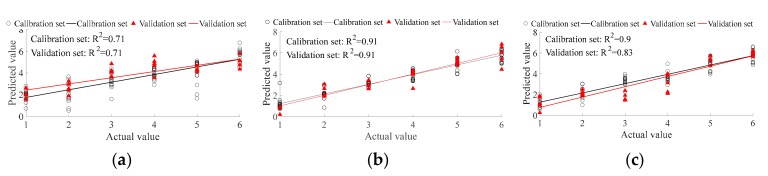

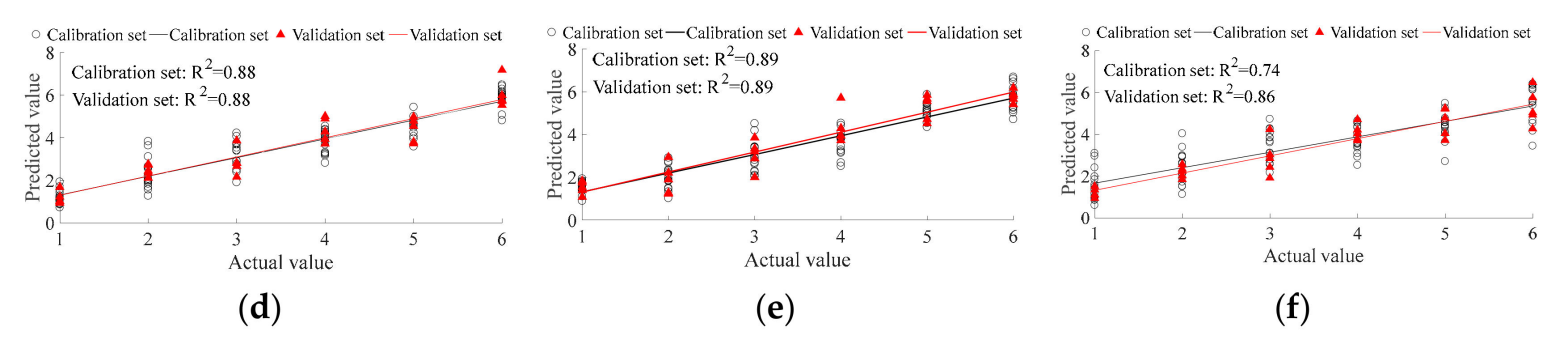
Partial least square regression (PLSR) for ancient Pu’er tea discrimination based on E-nose. (**a**–**c**) are traditional feature extraction; (**d**–**f**) are CNN feature extraction; (**a**,**d**) are E-nose detection; (**b**,**e**) are VIS/NIR spectrometer detection; and, (**c**,**f**) are multi-information fusion detection.

PLSR was applied for predictionto further compare the ability of different methods in the detection of ancient Pu’er tea type, and to find the most appropriate method among them. In this study, there were six tea types, with 20 replicates per type. Fifteen replicates of each type were randomly selected as a calibration set; the remaining five replicates formed the validation set. Therefore, there are 90 replicates in the calibration set, and 30 replicates in the validation set in total. The expected output of BM, HK, LBZ, LME, NK, and PS were set as 1, 2, 3, 4, 5, and 6, respectively. For PLSR results, fitting the correlation coefficient (R^2^) is the key parameter in evaluating the correlation between the predicted value and actual value. The range of R^2^ is from 0 to 1, where the greater the R^2^, the better the prediction ability. The PLSR type prediction ability that is based on E-nose and CNN feature extraction (Figure 4d) is better than that based on E-nose and traditional feature extraction (Figure 4a). The PLSR type prediction ability based on VIS/NIR spectrometer and traditional feature extraction (Figure 4b) is slightly better than that based on VIS/NIR spectrometer and CNN feature extraction (Figure 4e). The PLSR type prediction ability that is based on fusion data and traditional feature extraction (Figure 4b) is obviously better than that based on fusion data and CNN feature extraction (Figure 4e). For traditional feature extraction, the prediction ability, from the best to the worst, are VIS/NIR spectrometer, multi-source information fusion, and E-nose. For CNN feature extraction, the prediction ability, from the best to the worst, are VIS/NIR spectrometer, E-nose, and multi-source information fusion. For ancient Pu’er tea type prediction, the VIS/NIR spectrometer and traditional feature extraction method has the best ability (R^2^ of both calibration and validation set are equal to 0.9).

### 3.2. Blended Ratio Detection of Pu’er Tea

#### 3.2.1. LDA Classification of Pu’er Tea’s Blended Ratio

**Figure 5:**
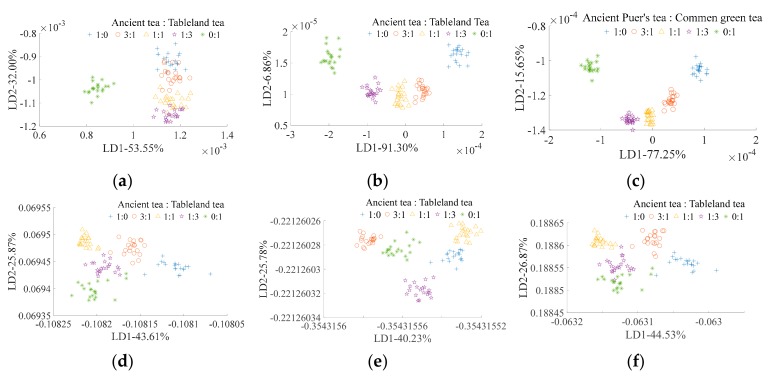
LDA for ancient Pu’er tea discrimination based on E-nose. (**a**–**c**) are traditional feature extraction; (**d**–**f**) are CNN feature extraction; (**a**,**d**) are E-nose detection; (**b**,**e**) are VIS/NIR spectrometer detection; and, (**c**,**f**) are multi-information fusion detection.

Figure 5a shows the LDA classification result of blended tea ratio (ancient tea:tableland tea) based on E-nose and traditional feature extraction. No tested blended ratios can be distinguished, except 0:1 (pure tableland tea). The LDA classification result of blended tea ratio based on E-nose and CNN feature extraction is shown in Figure 5d. All of the tested ratio blends can be categorized; however, the clustering of each ratio is inferior (the sample points distribution is more scattered) than categorization based on E-nose and traditional feature extraction (Figure 5a). The VIS/NIR spectrometer with both traditional (Figure 5b) and CNN (Figure 5e) feature extraction can efficiently classify the blended tea ratios; however, the clustering, when using the traditional feature extraction method, is slightly better than when using the CNN feature extraction method. Multi-source information with both traditional (Figure 5c) and CNN (Figure 5f) feature extraction can efficiently classify all of the tested blended tea ratios; however, the clustering ability of the traditional feature extraction method is obviously better than that of the CNN feature extraction method, as the group distances between the ratios of the traditional feature extraction method are larger than that of CNN feature extraction method. For traditional feature extraction, the blended tea ratios can be classified by both VIS/NIR spectrometer and multi-source information; however, multi-source information possesses better clustering ability than the VIS/NIR spectrometer. For CNN feature extraction, the VIS/NIR spectrometer has the superior ability to categorize the blended tea ratios. Among all of the classification methods in this study, multi-source information with traditional feature extraction was the most efficient method to determine the blend ratio of Pu’er with other types of tea. 

#### 3.2.2. PLSR Prediction of Pu’er Tea’s Blended Ratio

**Figure 6:**
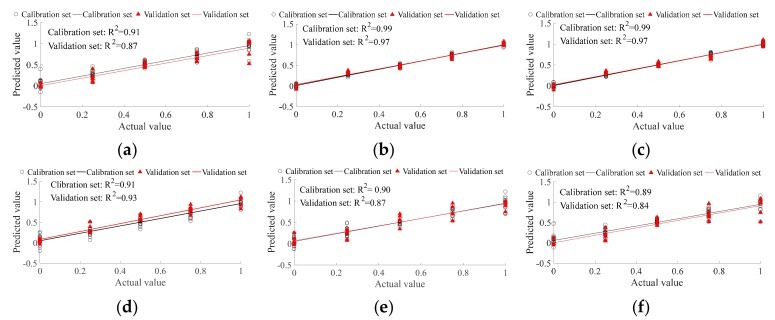
PLSR for ancient Pu’er tea discrimination based on E-nose. (**a**–**c**) are traditional feature extraction; (**d**–**f**) are CNN feature extraction; (**a**,**d**) are E-nose detection; (**b**,**e**) are VIS/NIR spectrometer detection; and, (**c**,**f**) are multi-information fusion detection.

PLSR was applied for predictionto further compare the ability of the various methods to detect the ratio of blended Pu’er tea and determine the most efficient method among them. In this study, there were five blended ratios, and each ratio had 20 replicates. Fifteen replicates of each ratio were selected randomly as the calibration set, while the remaining five replicates were used as the validation set. There were 75 replicates in the calibration set and 25 replicates in the validation set in total. The occupancy volume of tableland tea was set as the expected output of PLSR prediction. Thus, the expected output of the different ratios 1:0, 3:1, 1:1, 1:3, and 0:1 (ancient tea:tableland tea) were 0, 25%, 50%, 75%, and 100%, respectively. The prediction ability that is based on E-nose and CNN feature extraction (Figure 6d) is better than that based on the E-nose and traditional feature extraction (Figure 6a). The prediction ability based on VIS/NIR spectrometer and CNN feature extraction (Figure 6e) is worse than that based on VIS/NIR and traditional feature extraction (Figure 6b). The prediction ability that is based on fusion information and CNN feature extraction (Figure 6f) is worse than that based on fusion information and traditional feature extraction (Figure 6c). For traditional feature extraction, VIS/NIR spectrometer and fusion information has the same better prediction ability for blended ratio than E-nose. For CNN feature extraction, the E-nose has better prediction ability for the blended ratio than VIS/NIR spectrometer, and the blended ratio prediction ability based on fusion information is the worst. For all prediction methods in this study, both the VIS/NIR spectrometer and fusion information with traditional feature extraction had the best ability (R^2^ of both calibration and validation set are larger than 0.96) for Pu’er tea’s blended ratio prediction.

### 3.3. Mixed Ratio Detection of Pu’er Tea

#### 3.3.1. LDA Classification of Pu’er Tea’s Mixed Ratio

**Figure 7:**
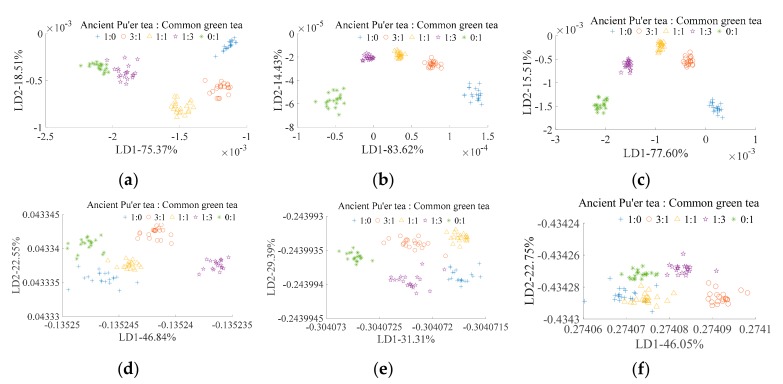
LDA for ancient Pu’er tea discrimination based on E-nose. (**a**–**c**) are traditional feature extraction; (**d**–**f**) are CNN feature extraction; (**a**,**d**) are E-nose detection; (**b**,**e**) are VIS/NIR spectrometer detection; and, (**c**,**f**) are multi-information fusion detection.

Figure 7a shows the LDA classification result of Pu’er tea’s mixed ratio based on E-nose and traditional feature extraction. All of the mixed ratios can be classified. Figure 7d shows the LDA classification result of Pu’er tea’s mixed ratio based on E-nose and CNN feature extraction. While all of the mixed ratios can be classified using this method, and the clustering ability is inferior than E-nose and traditional feature extraction (Figure 7a), especially for mixed ratios of 1:0, 3:1, and 0:1 (Pu’ertea:common green tea). The mixed ratios can be classified by VIS/NIR spectrometer with both traditional (Figure 7b) and CNN (Figure 7e) feature extraction methods; however, the clustering ability using the traditional method is superior to when using the CNN method. All of the mixed ratios can be classified by multi-source information and traditional feature extraction (Figure 7c), but not by multi-source information and CNN feature extraction (Figure 7f), and the clustering ability of the classification results from the former method (Figure 7c) is superior to that when using the later method (Figure 7f). For traditional feature extraction, the classification abilities of VIS/NIR spectrometer and multi-source information are better than those of an E-nose. For CNN feature extraction, the classification ability of the E-nose and VIS/NIR spectrometer are better than for multi-source information. Among all of the classification methods studied, VIS/NIR spectrometer and multi-source information with traditional feature extraction was the optimal method for Pu’er tea’s mixed ratio detection.

#### 3.3.2. PLSR Detection of Pu’er Tea’s Mixed Ratio

**Figure 8:**
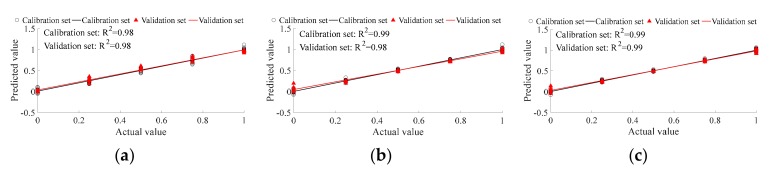

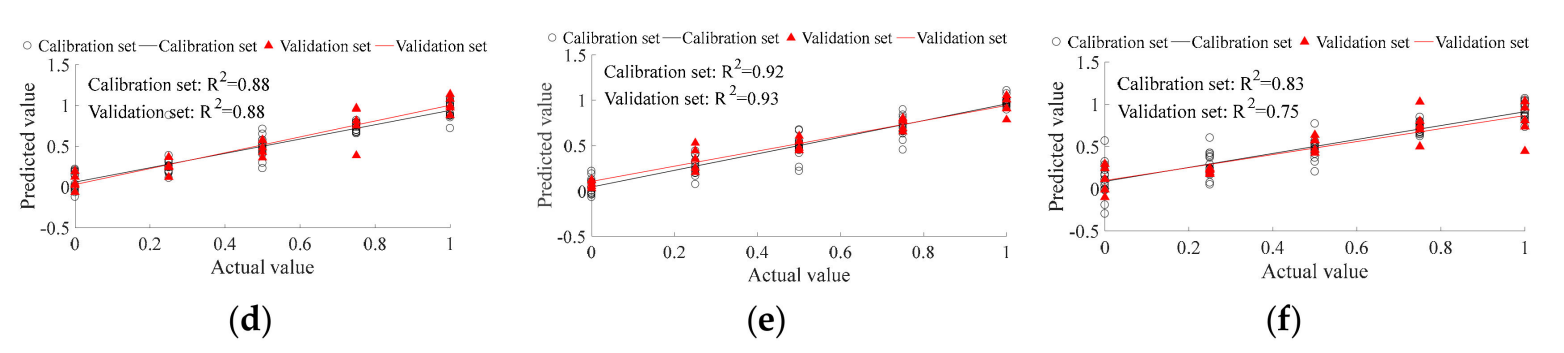
PLSR for ancient Pu’er tea discrimination based on E-nose. (**a**–**c**) are traditional feature extraction; (**d**–**f**) are CNN feature extraction; (**a**,**d**) are E-nose detection; (**b**,**e**) are VIS/NIR spectrometer detection; and, (**c**,**f**) are multi-information fusion detection.

PLSR was applied for prediction to further compare the abilities of the various mixed tea ratio detection methods and to determine the most efficient method among them. In this study, there were five mixed ratios, and each ratio had 20 replicates. Fifteen replicates of each ratio were randomly selected for the calibration set, with the remaining five replicates being used for the validation set. Therefore, there were 75 replicates in the calibration set and 25 replicates in the validation set in total. The occupancy volume of common green tea of each sample was set as the expected output; therefore, the expected output of different ratios 1:0, 3:1, 1:1, 1:3, and 0:1 (ancient tea:tableland tea) were 0, 25%, 50%, 75%, and 100%, respectively. The prediction ability of E-nose with traditional feature extraction (Figure 8a) is better than that with CNN feature extraction (Figure 8d). The prediction ability of the VIS/NIR spectrometer with traditional feature extraction (Figure 8b) is better than that with CNN feature extraction (Figure 8e). The prediction ability of multi-source information with traditional feature extraction (Figure 8b) is better than that with CNN feature extraction (Figure 8e). Multi-source information fusion can improve the prediction ability of single detection tools for traditional feature extraction. However, CNN feature extraction reduced the prediction ability of single detection tools. The optimal mixed tea ratio prediction method was multi-source information with traditional feature extraction (R^2^ of both calibration and validation set are equal to 0.99).

## 4. Discussion

### 4.1. Difference of Pu’er Tea among Types, Blended Ratios and Mixed Ratios

It has been previously proven that it is feasible to detect tea quality via E-nose or spectrometer [13,16]. However, previous researches mostly focused on the quality differences among teas from different provinces or cities [31], and focused less on the quality differentiation among teas from the same city, like different expensive varieties of ancient Pu’er teas from the city of Pu’er in Yunnan province. Previous research found that different Pu’er tea types display differences in aroma, taste, and chemical components (caffeine, amino acid, polyphenols, etc.) [32], which provide a theoretical basis for using an E-nose or a VIS/NIR spectrometer to determine an ancient Pu’er tea’s specific type, the ratio at which ancient Pu’er tea is blended with non-ancient Pu’er tea, or the ratio at which Pu’er tea is mixed with non-Pu’er tea. This study further indicated that an E-nose (Table 3) and a VIS/NIR spectrometer (Figure 1) can efficiently measure the characteristic differences between different types of ancient Pu’er tea, as well as between different ratios of blended and mixed teas (Figure 3, Figure 4, Figure 5, Figure 6, Figure 7 and Figure 8).

### 4.2. Different Feature Extraction Ways Affect Detection Result

CNN, as a deep learning method, has been proven to have strong feature extraction ability for image and speech signal processing, but less research has focused on CNN application for feature extraction of E-nose and spectrum data. Previous research of CNN applied to the feature extraction of E-nose and spectrum data showed better ability than the traditional method [33,34], but only slightly addressed the defects of CNN. In this study, CNN improved the LDA classification and the PLSR prediction ability of type and blended ratio detection based on E-nose data, but reduced the LDA classification and PLSR prediction ability of the type, blended ratio, and mixed ratio based on VIS/NIR spectrometer data and fusion information, as well as the mixed ratio classification based on E-nose data. The explanation for this decrease in ability could be that, while the CNN method can acquire a sample’s local feature comprehensively, not all of the detected local features are useful. Redundant information can decrease the detection ability (such as reduced the clustering ability of LDA and decreased the R^2^ of PLSR in this study). Unlike CNN, traditional extraction focuses on fewer and more relatively obvious features, which therefore results in more specific and less redundant data. When compared with the traditional method, and depending on the data source, CNN can both improve and reduce the detection ability. When the traditional method can collect enough sample features to support an accurate detection, the detection ability will be superior to the CNN feature extraction. However, the strong comprehensive local feature extraction ability of CNN may help to improve the detection ability when the traditional method is unable to collect sufficient sample feature data to support an accurate detection.

### 4.3. Different Detection Tools Affect Detection Result

E-nose, VIS/NIR spectrometer, and multi-source information fusion were applied in this study for ancient Pu’er tea quality detection. The analysis results of LDA and PLSR showed that the VIS/NIR spectrometer has better ability than E-nose for type, blended ratio, and mixed ratio detection of Pu’er tea. From this, we can infer that the Pu’er tea types, blends of different ratios, and mixes of different ratios differed more in material composition than in volatile profile. Multi-source information fusion can efficiently improve the detection ability of a single detection method [35], such as blended ratio detection using the traditional feature extraction method. However, multi-source information fusion maintains high risk of introducing redundant information to the data set [36]. Thus, multi-source information fusion insignificantly altered the detection ability for blended tea while using the traditional feature extraction method. The reason for this insignificant change might be due to the fact that single detection tools do notcomplement each other or introduce redundant data. Additionally, multi-source information fusion reduced the detection ability of type detection using both traditional and CNN feature extraction methods, and reduced the detection of ability for blended and mixed teas when using the CNN feature extraction method. Again, the presumed reason for this is the introduction of redundant information.

### 4.4. The Suggested Method in Practical Detection

According to our results, when considering the operation costs (from the most to the least expensive: multi-source information fusion > E-nose > VIS/NIR spectrometer) and the average time consumption (from the longest to the shortest time expenditure: multi-source information fusion > VIS/NIR spectrometer > E-nose for detection tool, and CNN > traditional for extraction method) into consideration, Pu’er tea type detection should be conducted using the VIS/NIR spectrometer and traditional feature extraction, which has superior accuracy relative to the other methods. Both VIS/NIR spectrometer and multi-source information fusion with traditional feature extraction have almost the same ability for blended ratio detection; however, the cost and time consumption of multi-source information fusion is higher than the VIS/NIR spectrometer. Therefore, blended tea should be analyzed using VIS/NIR spectrometer with traditional feature extraction. Both the VIS/NIR spectrometer and multi-source information fusion with traditional feature extraction can efficiently detect and categorize mixed tea, with the detection ability of multi-source information fusion being slightly better than VIS/NIR spectrometer. However, when considering the cost and time consumption, the detection method for mixed tea should be predicated on specific conditions. Multi-source information fusion with traditional feature extraction should be used if the accuracy requirement is extremely high, while VIS/NIR spectrometer with traditional feature extraction is otherwise preferred.

## 5. Conclusions

This experiment used an E-nose and a VIS/NIR spectrometer to sample ancient Pu’er tea in six types, five blended ratios, and five mixed ratios. Traditional and CNN methods were applied for the feature extraction of E-nose and VIS/NIR spectrometer sampling data. LDA and PLSR were used for pattern recognition. The experimental results are as follows.

(1) For E-nose and VIS/NIR spectrometer data of Pu’er tea that was extracted using the traditional method, the mean differential value of each sensor should be selected as the optimal feature extraction method for E-nose data. The 594, 618, 639, 672, 726, 1107, 1196, 1300, 1432, 1472, 1631, 1727, 1852, 1928, 2015, 2140, 2218, 2308, and 2324 nm values and two slope values during 726 to 1107 nm and 1300 to 1432 nm, should be selected as VIS/NIR spectrometer feature, for Pu’er tea type, blended ratio, and mixed ratio detection.

(2) The LDA and PLSR analysis results showed that CNN could acquire more local features than the traditional method to improve the detection ability, but with the risk of including redundant information, which reduces the detection ability.

(3) Multi-source information fusion (E-nose and VIS/NIR spectrometer data fusion) can collect more features from different angles to improve the detection ability, but it also has the risk of adding redundant information, which reduces the detection ability.

(4) For practical detection, the Pu’er tea type should be detected by using a VIS/NIR spectrometer and traditional feature extraction. The blended ratio of Pu’er tea should be detected by using VIS/NIR spectrometer with traditional feature extraction. Multi-source information fusion with traditional feature extraction should be applied if the accuracy requirement is extremely high for mixed ratios; otherwise, the VIS/NIR spectrometer with traditional feature extraction is preferred.

(5) The results of this experiment provide a quick and accurate method for detecting the type, blend ratio, and mix ratio of Pu’er tea, which is significant in maintaining the integrity of the Pu’er tea industry.

## Figures and Tables

**Table 1:** Sample information of experimental Pu’er tea.

	Ancient Pu’erTea								TABLELAND TEA	Non-Pu’erTea
**Type**	LBZ ^a,x^	LME ^a,x^	HK ^a,x^	PS ^a,x^	BM ^a,x^	NK ^a,x^	YW ^b,x^	BP ^c,x^	YW ^b,x^	QLS ^c,y^

Note: ^a^ for type test; ^b^ for blended ratio test; ^c^ for mixed ratio test; the production year and place were: ^x^ 2017/Yunnan; y. 2017/Guangdong.

**Table 2:** Sensor name and performance of electronic nose (PEN3).

Number in Array	Sensor Name	Object Substances for Sensing	Threshold Value (mL·m^−3^)
R1	W1C	Aromatics	10
R2	W5S	Nitrogen oxides	1
R3	W3C	Ammonia and aromatic molecules	10
R4	W6S	Hydrogen	100
R5	W5C	Methane, propane and aliphatic non-polar molecules	1
R6	W1S	Broad methane	100
R7	W1W	Sulfur-containing organics	1
R8	W2S	Broad alcohols	100
R9	W2W	Aromatics, sulfur-and chlorine-containing organics	1
R10	W3S	Methane and aliphatics	10

**Table 3:** Analysis of variance (ANOVA) for feature difference analysis.

Features	The *p* Values of Different Discrimination Targets		
	Type	Blended Ratio	Mixed Ratio
Maximum	0.06	0.30	1.45 × 10^−11^
Mean differential value	0.01	0.04	1.31 × 10^−11^
Average value	0.03	0.42	3.49 × 10^−11^
The 95 s value	0.23	0.83	2 × 10^−4^

**Table 4:** Paremeter setting of CNN feature extraction.

		CNN Network Structure						Learning Rate	Iterations
		Layer 1			Layer 2				
		FMN	FMS	PS	FMN	FMS	PS		
Type	E-nose	10	2	3	16	2	3	0.75	1000
	VIS/NIR	8	5	2	14	5	3	0.55	1000
Blended ratio	E-nose	12	3	2	18	2	3	0.70	1000
	VIS/NIR	21	3	4	12	2	2	0.60	1000
Mixed ratio	E-nose	6	2	3	8	2	3	0.60	1000
	VIS/NIR	10	3	2	16	3	3	0.55	1000

Note: FMN is the number of feature map; FMS is the size of feature map; PS is the size of the pooling layer.
